# Effect of monothiophosphinic acid and phosphine sulfide impurities on the solvent extraction behavior of bis(2,4,4-trimethylpentyl)dithiophosphinic acid from sulfate and methanesulfonate media

**DOI:** 10.1039/d5ra06902e

**Published:** 2026-01-05

**Authors:** Tim Balcaen, Koen Binnemans, Stijn Raiguel

**Affiliations:** a KU Leuven, Department of Chemistry Celestijnenlaan 200F, P.O. Box 2404 B-3001 Leuven Belgium stijn.raiguel@kuleuven.be

## Abstract

A crude, synthesized mixture of bis(2,4,4-trimethylpentyl)dithiophosphinic acid (DTPhos), was evaluated as a functional alternative to the discontinued Cyanex® 301 extractant. This study enabled the assessment of the influence of common impurities, such as bis(2,4,4-trimethylpentyl)monothiophosphinic acid (MTPhos) and phosphine sulfides, on metal extraction performance from methanesulfonate (MSA) and sulfate media. Both extractants exhibited nearly quantitative extraction of Ni(ii), Co(ii), and Zn(ii) under non-saponified conditions. In contrast to Cyanex 301, synthesized DTPhos-based mixtures did not give rise to third-phase formation at elevated pH values in methanesulfonate media. Additionally, Mn(ii) was extracted at lower pH values with the DTPhos mixtures than with Cyanex 301. Regression analysis of distribution ratios confirmed comparable cobalt(ii) extraction efficiencies and revealed a moderate reduction in separation from nickel(ii) for the least-enriched DTPhos formulation. Overall, the effect of the DTPhos impurity on the extraction properties was limited under the investigated conditions. Furthermore, variations in extraction mechanisms were suggested by pH-dependent behavior, particularly in sulfate-based systems. These findings support the use of synthesized DTPhos as a viable alternative to Cyanex 301, offering practical benefits such as stability against third-phase formation and flexibility in extractant composition.

## Abstract 2

In-depth study of the extraction behavior of commercial *versus* synthesized dithiophosphinic acid extractants reveals significant differences in Co(ii)/Ni(ii)/Mn(ii) selectivity and the physical stability of the system.

## Graphical abstract

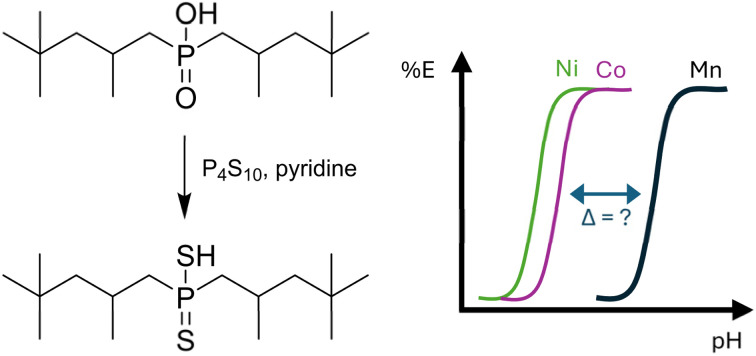

## Introduction

**Fig. 1:**
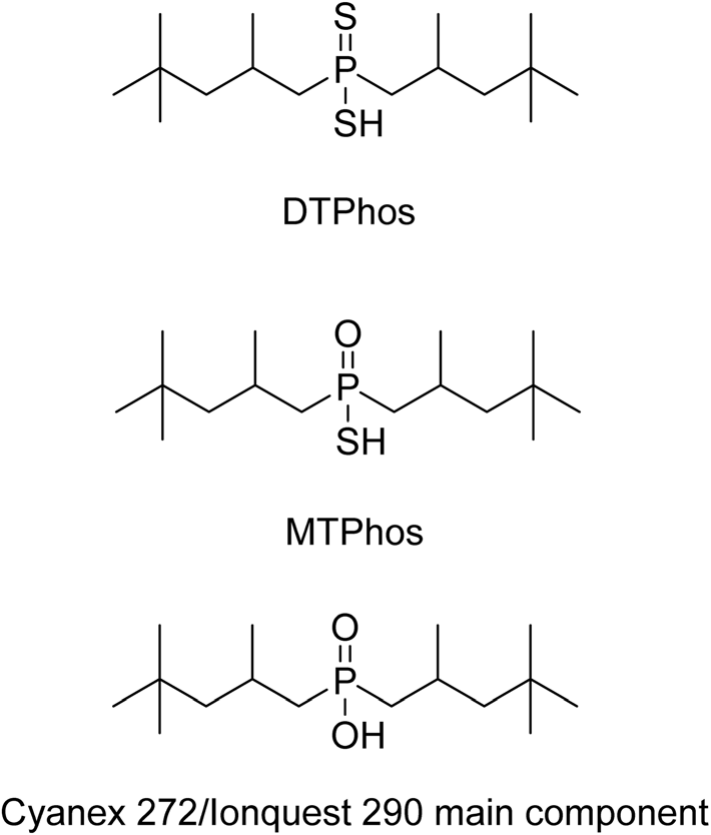
Structures of the acidic extractants discussed in this text: bis(2,4,4-trimethylpentyl)dithiophosphinic acid (DTPhos, upper), bis(2,4,4-trimethylpentyl)monothiophosphinic acid (MTPhos, lower) and bis(2,4,4-trimethylpentyl)phosphinic acid (lower), the main component of Cyanex 272 and Ionquest 290.

In cobalt and nickel extractive metallurgy, cobalt- and nickel-rich laterite ores are usually leached using strong mineral acids, after which the solution is neutralized to yield a mixed hydroxide precipitate (MHP) or mixed sulfide precipitate (MSP).^1–3^ Subsequently, this precipitate is redissolved to allow separation and purification of the transition metals by solvent extraction, using organophosphorus and carboxylic acids as extractants. These extractants will only extract cobalt and nickel at relatively high pH values (4–7), which precludes direct extraction of cobalt and nickel from acid leachates without prior neutralization of the excess acid in the leaching liquor. Alternatively, cobalt and nickel can be recovered from the strongly acidic leach liquor directly using Cyanex® 301, a strongly acidic extractant containing a dithiophosphinic acid as the main constituent (*i.e.* bis(2,4,4-trimethylpentyl)dithiophosphinic acid, Fig. 1). This extractant can hence be seen as the dithio analog of Cyanex 272 (mainly bis(2,4,4-trimethylpentyl)phosphinic acid, Fig. 1), the extractant commonly used to separate cobalt and nickel in hydrometallurgical flowsheets.^3,4^

Cyanex 301 can extract Zn(ii), Co(ii) and Ni(ii) from solutions with a pH of 1 or lower, which is in stark contrast to the pH values required for Cyanex 272 (pH 2–7).^3^ This difference is mainly attributed to the higher acidity of the dithiophosphinic acid moiety.^5^ Mn(ii) is only extracted at higher pH, allowing the preferential extraction of Co(ii) and Ni(ii) from the less valuable Mn(ii), a property not shared with any other commercial extractant.^3,6,7^ Dimerization of the extractant only occurs at higher concentrations, unlike Cyanex 272, which always exists as a hydrogen-bonded dimer.^4,8^ Mechanistic studies have also revealed that Cyanex 301 forms with Co(ii) and Ni(ii) complexes with a simple 1 : 2 stoichiometry, while the species extracted by Cyanex 272 can either contain extractant dimers in the first coordination sphere, or form polymeric structures or aggregates.^4,9^ In addition to its use in cobalt and nickel hydrometallurgy and some other minor applications, Cyanex 301 has also been extensively studied as an extractant for the separation of americium from lanthanide fission products, with potential applications in spent nuclear fuel reprocessing. Here, the soft nature of the sulfur donor atoms confers selectivity to the extractant for Am(iii).^10–12^

Cyanex 301 was originally developed and produced by Cytec (product lien currently owned by Syensqo), but it is no longer commercially available. Similar formulations are still being produced by some Chinese manufacturers, but it no longer appears to be possible to procure samples outside of the People's Republic of China, due to legislation pertaining to the transport of hazardous chemicals.

On the other hand, two formulations are commercially available with bis(2,4,4-trimethylpentyl)phosphinic acid as main component, which is the oxygen analog of the main component of Cyanex 301: (1) Cyanex 272, which contains significant amounts of phosphine oxide impurities (supplier: Syensqo), and (2) Ionquest 290, which is of higher purity (supplier: Italmatch). Cyanex 272 has been applied to the separation of cobalt and nickel in both research and industrial settings for several decades, and new flowsheets continue to be developed.^13–15^ As a number of synthetic methods exist to convert P–O bonds to P–S bonds, a formulation similar to Cyanex 301 can thus be produced for research purposes from commercially available phosphinic acids as starting materials. These conversions make use of the Berzelius or the Bergman reagents.^16–21^

Therefore, we have evaluated a multi-gram synthetic approach to convert Ionquest 290 into, and studied the influence of side products and impurities on the extraction performance on the resulting formulation.^22,23^ Both commercial Cyanex 301 and synthesized formulations contain a number of impurities, including some which act as modifiers, synergists, antagonists or even as extractants by themselves. These impurities have been reported to affect the separation of lanthanides and actinides by Cyanex 301. The primary goal of this study was to assess the impact of certain impurities on the extraction performance of bis(2,4,4-trimethylpentyl)dithiophosphinic acid formulations, both in terms of selectivity and phase separation.

## Materials and methods

### Chemicals and reagents

Phosphorus pentasulfide (99%, Merck life science BV), Ionquest® 290 (95%, Italmatch), pyridine (certified AR for analysis, Fisher Scientific BV), molecular sieves (type 3 Å, Fischer Scientific BV), hydrochloric acid (37% analytical reagent grade, Acros Organics NV), isohexane (<3 ppm residue, Acros Organics NV), heptane fraction from petroleum (Laboratory reagent grade, Fisher Scientific BV), sodium hydrogen carbonate (analytical reagent grade, Acros Organics NV), methanol (certified AR for analysis, Fischer Scientific BV), 1-butanol (Emsure®, Merck life science BV), deuterium oxide (for NMR, 99.8 atom% D) and orthophosphoric acid (analytical reagent grade), methanesulfonic acid (≥99.5%, Carl Roth GMBH), *n*-dodecane (TCI Europe NV), nickel(ii) carbonate, basic hydrate (99.9%, Merck life science BV), zinc(ii) carbonate basic (97.0%, Thermo Fisher (Kandel) GMBH), cobalt(ii) carbonate (43–47% Co, Merck life science BV), manganese(ii) carbonate (99.9%, Merck life science BV), basic magnesium carbonate (heavy, Merck life science BV), calcium carbonate (99%, Carl Roth GMBH), nitric acid (65 wt% VWR International BV), sodium hydroxide (analytical reagent grade, Fisher Scientific BV), multielement Standard Solution 6 for ICP (TraceCERT®, VWR International BV), nickel(ii) sulfate hexahydrate (p.a., Th. Geyer GMBH), manganese(ii) sulfate monohydrate (a.r., Chem-Lab NV), calcium sulfate dihydrate (a.r., Chem-Lab NV), cobalt(ii) sulfate heptahydrate (99+%, Acros Organics BV), zinc(ii) sulfate monohydrate (99%, Acros Organics BV), magnesium sulfate (dried, VWR International BV). Cyanex® 301 was purchased from Solvay NV (Brussels, Belgium).

All chemicals were used as received. Ultrapure water was generated using a Millipore Milli-Q Reference® system, purified to a total organic content (TOC) of <2 ppb and a resistivity of 18.2 MΩ cm.

### Instrumentation

Inductively coupled plasma-optical emission spectroscopy (ICP-OES) was used for the analysis of cobalt, nickel, manganese, calcium, magnesium and zinc in the aqueous phase. ICP-OES analyses were carried out using a PerkinElmer Avio 500 equipped with a GemCone high solids nebulizer, baffled cyclonic spray chamber, 2.0 mm inner diameter alumina injector and a PerkinElmer Hybrid XLT torch. Samples for the quantification of cobalt, nickel, zinc, calcium, magnesium and manganese were prepared diluting the aqueous phase with a 2 vol% solution of HNO_3_ in ultrapure water to obtain metal concentrations within the calibrated range. External calibration curves were constructed with a calibration series containing 0, 0.1, 0.2, 0.5, 1, 5, 10, 20 and 30 mg L^−1^ of above-mentioned elements. The plasma, auxiliary and nebulizer gas flows were set to 8 L min^−1^, 0.2 L min^−1^ and 0.6 L min^−1^, respectively. Each dilution was measured in triplicate, in axial mode for manganese and radial mode for nickel, cobalt, magnesium, calcium and zinc. Spectral lines 231.604 nm, 228.616 nm, 257.61 nm, 285.213 nm, 317.933 nm and 206.2 nm were chosen for nickel, cobalt, manganese, magnesium, calcium and zinc, respectively. Measurement of quality control standard samples revealed the results to be accurate to within 5% of the expected concentration.

High-field nuclear magnetic resonance (NMR) spectra were recorded on a Bruker Avance III HD 400 spectrometer with a Bruker AscendTM 400 magnet system (^1^H basic frequency of 400.17 MHz) and a 5 mm PABBO BB/19F-1H/D probe with z-gradients or on a Bruker Avance II+ 600 spectrometer with a Bruker 600 UltraShieldTM magnet system (1H basic frequency of 600.13 MHz) and a 5 mm PABBO BB-1H/D probe with z-gradients. ^31^P-detected experiments were measured using inverse-gated broadband decoupling, 30° pulse angle, NS = 4, SW F1/F2 = 170, O1P = 50, D1 = 30. Pulse program: zgig30. All samples were diluted with chloroform-*d* (CDCl_3_) or were measured undiluted (external reference containing D_2_O for locking). Data were recorded at room temperature using Bruker TopSpin 4.1.3. ^31^P NMR spectra were calibrated based on a dilution of 10% orthophosphoric acid solution in D_2_O. The phosphoric acid signal was calibrated at 0 ppm. The *δ*-values are expressed in parts per million (ppm). The following acronyms were used: s (singlet), d (doublet), m (multiplet), brd (broadened).

LR-MS data was recorded using a Waters Radian ASAP MS probe. The apparatus uses single quadrupole mass spectrometry technology (mass range: *m*/*z* 30–1250) combined with the Atmospheric Pressure Solids Analysis Probe (ASAP). Spectra were obtained in both positive and negative ionization mode (APCI, corona current: 3(+)/6(−) µA, cone voltage: +15/−20 V, source temperature 150 °C, gas heater temperature 450 °C). Data were acquired on a computer using MassLynx software and further analysis was done using ACD/Spectrus Processor 2022.1.6.

FT-IR spectra were recorded on a Bruker Vertex 70 infrared spectrometer, equipped with Platinum ATR module and diamond sample crystal. Data were analyzed using the OPUS software package.

pH measurements were performed using a FiveEasy pH meter F20 (METTLER TOLEDO) with pH microelectrodes (InLab® Micro).

### Procedure for solvent extraction

The solvent extraction experiments were performed in 4-mL glass vials. The organic phase was prepared by diluting stock solutions of either Cyanex 301 or the synthesized mixture of MTPhos and DTPhos in *n*-dodecane. The extractant concentration was chosen such that the sum of the concentration of MTPhos and DTPhos is within the same order of magnitude for all four organic phases (Table 2). Additionally, the extractant concentration was at least four times larger than the total concentration of metal ions (Table 1). Extractant solutions were prepared gravimetrically for increased accuracy.

**Table 1:** Concentration of metal ions of stock solutions for the methanesulfonate (MSA) and sulfate medium. The concentrations were determined *via* ICP-OES

Element	*M*_MSA medium_ (mmol L^−1^)	*M*_sulfate medium_ (mmol L^−1^)
Ni	42.14	53.11
Co	20.87	21.69
Mn	19.07	20.91
Mg	45.25	38.34
Ca	50.56	6.36
Zn	10.01	10.51
Total	187.90	150.92

Two metal stock solutions were prepared containing either the sulfate or methanesulfonate salts of Mg(ii), Mn(ii), Ni(ii), Co(ii), Zn(ii) and Ca(ii). The methanesulfonate salts were prepared *in situ* by reaction of the carbonate salts with stoichiometric amounts of methanesulfonic acid (MSA). Methanesulfonate media were investigated in addition to sulfate media as methanesulfonic acid has exhibited promising properties in (electro)metallurgy, such as enhanced solubility for certain metal salts and the formation of smooth deposits in electrodeposition without the need for additives to the electrolyte.^24^ The final compositions of both stock solutions can be found in Table 1. Note that the concentration of Ca(ii) ions is lower than that of other cations due to the poor solubility of CaSO_4_ in water. The total concentration of metal ions in both solutions was chosen such that it is at least smaller than four times the total concentration of extractant molecules given a total loading of extractant < 50%.

Within the aqueous phase, the metal stock solutions were diluted by Milli-Q water and NaOH (901.2 mM) such that saponification of the extractant ranges from 0% to 50%. Note that, due to the differences in extractant concentration in the organic phase, the true saponification differs slightly (±0.2%) from the expected saponification. The aqueous and organic phases were contacted for 24 h (25 °C, 2500 rpm). Afterwards, phase separation was facilitated by centrifugation. Phases were manually separated immediately after centrifugation. The pH of the aqueous phase was determined in all samples. This was followed by measurement of the metal concentrations by ICP-OES in the aqueous phase of samples that did not show any gel formation. The phase volume ratio was assumed to remain equal to 1 : 1, with 1 mL of each phase. Because of the limited available supply of the extractants, replicate samples were not produced, but errors were analyzed by statistical modelling of the data (*vide infra*).

### Calculation of percentage extraction and distribution ratio

The percentage of extraction is calculated according to eqn (1). Since the accuracy and precision of measurements are superior when performed on the aqueous phase, the remaining metal concentration is inferred by subtracting the concentration of metal in the aqueous phase from the concentration of metal in the stock solution.1
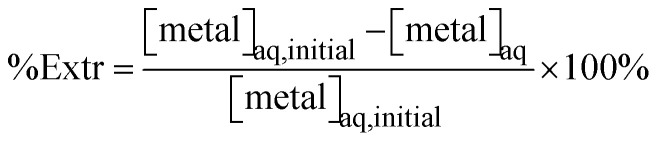


The same reasoning was applied to the computation of the distribution ratio (*D*, eqn (2)). In this formula, the organic-over-aqueous phase ratio, θ, is not explicitly written and assumed to equal 1, implying a phase ratio of 1 : 1.2
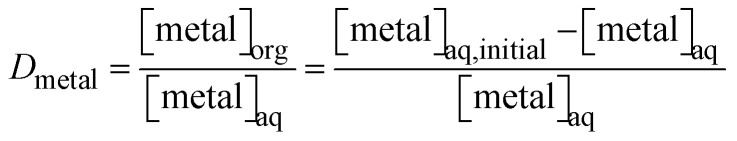


### Statistical analysis

Statistical analyses within the framework of the slope analysis or estimation of the logistic function were performed in JMP (version 17.2) and RStudio (2024.12.1). Prior to interpretation of estimated regression coefficients, model assumptions were verified by inspecting residual plots and the QQ plot. Derivation of the sigmoid function is given in the SI (SI, page S8).

## Results and discussion

### Multi-gram synthetic procedure for synthesis of crude bis(2,4,4-trimethylpentyl)dithiophosphinic acid

The synthetic procedure was based on a combination of patent literature^20^ and organic synthesis literature concerning the Berzelius^16,17^ and Bergman^18,19,21^ reagents. An approach using hexamethyldisiloxane (HMDSO) was also evaluated in initial experiments (data not reported), but this was not pursued further.^25^ While this reagent did reduce the formation of some aspecific side products, it also reduced the DTPhos/MTPhos ratio in the final product. Furthermore, the use of this reagent impacts the atom economy of the reaction. Ionquest 290 was selected as the starting material for the synthesis, as this extractant does not contain any phosphine oxide impurities, thus allowing the assessment of the influence of this impurity on extraction performance by comparison with Cyanex 301.

The synthesis was not optimized on larger scale and was executed twice with similar results. Small-scale optimization is outside of the scope of this article and is not representative of the results obtained on a larger scale.

Pyridine was dried overnight using activated molecular sieves (3 Å). A flame-dried, N_2_-purged 2 L three-necked round-bottom flask was fitted with two rubber stoppers, a reflux condenser, and a base trap on top of the reflux condenser. A H_2_S monitor was used for safety during the whole process. The flask was charged with a stirring bar and 265 g of P_4_S_10_ (596.1 mmol, 0.76 equiv.), followed by N_2_ purging. A N_2_-filled balloon was added to maintain an inert atmosphere. Dry pyridine (1 L) was slowly added under vigorous stirring, and the mixture was heated to 90 °C over a period of 20 min. After reaching 90 °C, 250 mL of Ionquest® 290 (788.5 mmol, 1 equiv.) was added slowly over 30 min. The reaction proceeded for 91 h 20 min, until no further product was formed, confirmed by ^31^P NMR spectroscopy (inverse-gated).

The reaction mixture was cooled to room temperature, then transferred to a 5-L flask for quenching with 3 M HCl (1–2 drops per s, 2 L) using an addition funnel. During quenching, the solution was stirred vigorously to prevent the formation of hotspots. The solid particles gradually dissolved and the mixture separated into two phases. Once the addition of HCl stopped producing gas or heat, extraction was performed. The mixture was extracted in 500 mL portions with *iso*-hexane (500 mL), and washed three times with 1 mol L^−1^ HCl (3 × 500 mL). If no clear phase separation was achieved, additional 1 mol L^−1^ HCl or 1-butanol was added. Phase samples were analyzed *via*^31^P NMR spectroscopy to ensure proper removal of most impurities.

The solvent was evaporated, yielding 207 g of crude material, containing approximately 45 g of bis(2,4,4-trimethyl)dithiophosphinic acid based on ^31^P NMR spectroscopy. Further purification involved solvent extraction: the crude product was diluted with *iso*-hexane (500 mL) and extracted six times with 80/20 V/V NaHCO_3_/MeOH (6 × 100 mL). The polar phase contained mono- and dithiophosphinic acids, while the apolar phase retained apolar and aprotic species. The aqueous phase was reacidified with 3 M HCl (500 mL) and extracted with *iso*-hexane (3 × 250 mL), with 50 mL of 1-butanol added for phase separation. The resulting mixture (53.72 g) appeared as a dark green viscous liquid consisted of approximately 68% bis(2,4,4-trimethyl)dithiophosphinic acid (DTPhos), 29% bis(2,4,4-trimethylpentyl)thiophosphinic acid (MTPhos) and 3% impurities. The final yield based on the ^31^P NMR purity estimation is 15.5% (36.5 g, 113.3 mmol). The final mixture was characterized by LR-MS (ASAP-MS) and ^1^H, ^13^C and ^31^P NMR. NMR spectra of DTPhos-enriched mixtures are provided (ESI, page S3).

LR-MS: bis(2,4,4-trimethylpentyl)dithiophosphinic acid: *m*/*z* calculated for (C_16_H_34_PS_2_^−^) 321.19; found: 321.2. ^1^H NMR (CDCl_3_, 600 MHz, ppm): ^1^H NMR (CDCl_3_, 600 MHz, ppm): 2.32 (1H, m), 2.2 (1H, m), 2.04 (1H, m), 1.44 (1H, dd, *J* = 14.02 Hz and 4.27 H2), 1.23 (1H, ddd, *J* = 2.47 Hz, 6.61 Hz and 14.04 Hz), 1.19 (3H, dd, *J* = 6.61 Hz and 1.09 Hz), 0.95 (9H, s). ^13^C NMR (CDCl_3_, 150 MHz, ppm): 55.22, 55.21, 55.14, 55.12, 51.46, 51.39, 51.14, 51.07, 33.58, 32.31, 32.30, 28.47, 28.44, 28.35, 28.32, 26.51, 26.48. ^31^P NMR (CDCl_3_, 243 MHz, ppm): 66.63, 66.53.

Density (25.00 °C): 0.9545 g cm^−1^.

Dynamic viscosity (25.00 °C): 279 mPa s.

After workup, extractant formulations were obtained containing mostly bis(2,4,4-trimethylpentyl)dithiophosphinic acid (DTPhos) and bis(2,4,4-trimethylpentyl)monothiophosphinic acid (MTPhos). The chemical structures of the compounds are shown in Fig. 1. Through successive workup steps, formulations were obtained with varying MTPhos content.

### Enrichment process of bis(2,4,4-trimethyl)dithiophosphinic acid

**Fig. 2:**
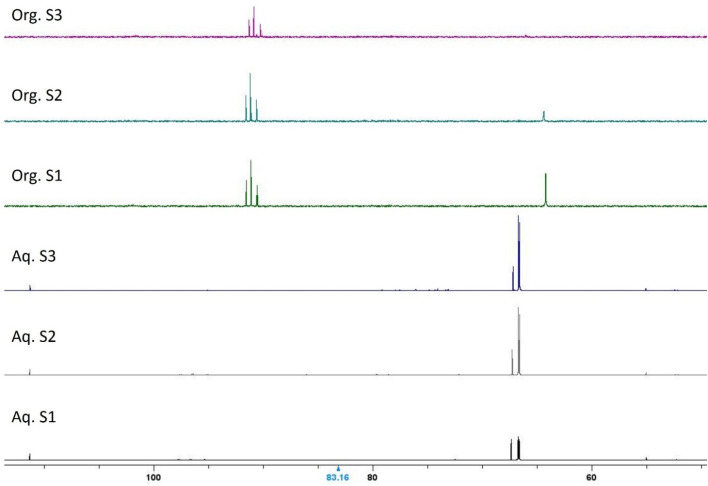
Representative ^31^P NMR spectra of the aqueous and organic phase during the extraction-based enrichment process (S1: 99 mM, S2: 156 mM and S3: 214 mM). Spectra are calibrated against a 10% H_3_PO_4_ in D_2_O external standard.

In order to obtain extractant formulations with varying concentrations of MTPhos impurity, a solvent extraction-based enrichment process was devised. This process was first optimized on a small scale using 4 mL glass vials. The crude DTPhos mixture was extracted using NaOH solutions of varying concentrations: 99 mM (Fig. 2 and S1), 156 mM (Fig. 2 and S2), and 214 mM (Fig. 2 and S3). In each sample, 2.4 mL of aqueous phase, consisting of 2.2 mL of deionized water and 0.2 mL of methanol, was mixed with 2.4 mL of organic phase, which consisted of 200 µL of 1-butanol, 200 µL of the crude DTPhos mixture (least pure), and 2 mL of heptane. After equilibration of the phases, the composition of the phases was analyzed using ^31^P NMR spectroscopy (Fig. 2). All DTPhos was extracted in sample S3, while extraction was incomplete in sample S2. However sample S3 also presented significant coextraction of MTPhos, and hence a base concentration intermediate between S2 and S3 was considered as optimal. Note that the presence of an unidentified signal in the aqueous phase ^31^P NMR spectra with a chemical shift close to that of DTPhos, which is completely extracted to the aqueous phase prior to full extraction of the DTPhos.

The optimized process was performed on 10 mL of the crude DTPhos mixture. The mixture was combined with 10 mL of 1-butanol and 100 mL of heptane to form the organic phase. The aqueous phase consisted of 7 mL of 1 mol L^−1^ NaOH, 93 mL of water, and 10 mL of methanol. The organic phase was extracted three times with the aqueous phase, and 1-butanol was added to ensure proper phase separation. The organic phase was discarded while the aqueous phase was then neutralized using 1 mol L^−1^ HCl and extracted three times with heptane. The combined heptane layers were washed three times with a methanol/water in 1 : 1 volume ratio. NMR analyses were performed on both the polar and apolar phases. This resulted in the most enriched fraction containing approximately 90 mol% DTPhos, 9 mol% MTPhos and 1 mol% other impurities.

A modified procedure was performed on 15 mL of the crude mixture was used, where a fourth NaOH extraction was introduced after the initial three extractions. This resulted in the medium enriched fraction containing approximately 85 mol% DTPhos, 14 mol% MTPhos and approximately 1 mol% impurities.

### Extractant composition

Four extractant solutions were made in total (Table 2). The solvent for all four is *n*-dodecane. To further improve accuracy of the experiment, all volumes were weighed and corrected by the measured or assumed density of the liquid extractant mixture. Important to note is that the synthesized extractant solutions were mainly composed of DTPhos and MTPhos, while the commercial Cyanex 301sample also contained impurities that could act as modifiers, such as tris(2,4,4-trimethylpentyl)phosphine sulfide (TOPS). A detailed analysis of the composition of Cyanex 301 and the synthesized mixtures is given in the SI (SI, page S4).

**Table 2:** Concentrations of MTPhos and DTPhos in the organic phases after dilution with *n*-dodecane, as determined by NMR spectroscopy

Mixture	Mol% DTPhos in extractant	Vol% extractant	*M*_DTPhos_ (mmol L^−1^)	*M*_MTPhos_ (mmol L^−1^)	*M*_Total_ (mmol L^−1^)
Synthesized (least enriched)	68	30	609.3	273.5	882.8
Synthesized (medium enriched)	85	30	753.6	143.3	896.9
Synthesized (most enriched)	90	30	807.3	93.4	900.7
Cyanex 301	68	41	809.3	50.1	859.4

### Comparison between Cyanex 301 and DTPhos in a synthetic MSA feed

In order to obtain insights in the performance of the crude synthesized mixture (Table 2, least enriched), the extractaction performance of this mixture was compared with that of commercial Cyanex 301 on a synthetic methanesulfonate (MSA) feed (Table 1), using *n*-dodecane as the diluent. No modifiers were added so that a fundamental evaluation of the behavior of both solutions was possible. This in turn resulted in mild to severe gel formation from 20% saponification onwards in the samples containing Cyanex 301 as extractant.

**Fig. 3:**
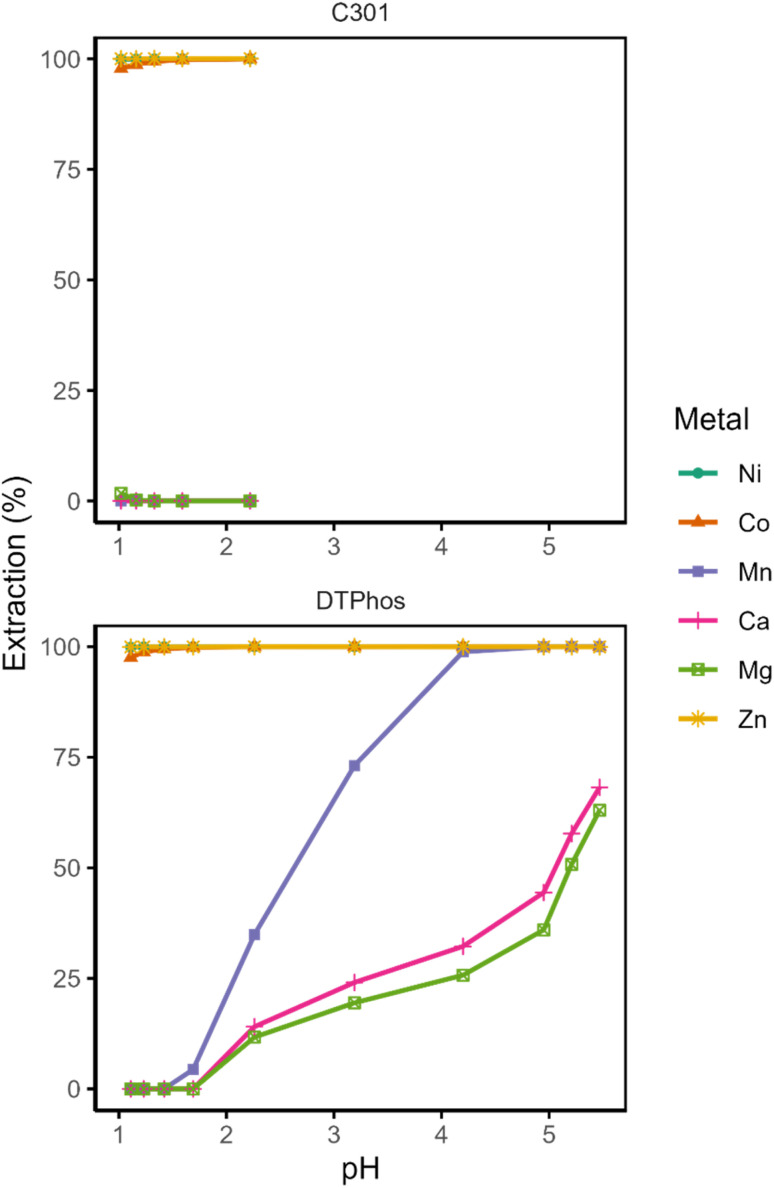
Equilibrium pH-extraction curves (22 °C) of the different metals extracted using Cyanex 301 (41 vol% in *n*-dodecane) and the synthesized, least enriched DTPhos mixture (30 vol% *n*-dodecane). Composition of the aqueous phase can be found in Table 1.

Afterwards, for all samples that did not contain a gel, the concentration of metals in the aqueous phase was measured by ICP-OES. Both extractant solutions were able to remove Ni(ii), Co(ii) and Zn(ii) nearly quantitatively after just one contact without saponification. Important differences arose upon saponifying the extractant (Fig. 3). Extraction of Mn(ii) was observed starting at an equilibrium pH of 1.69 in the DTPhos system, while no Mn(ii) was extracted in the commercial Cyanex 301 system until gel formation was observed (*i.e.* at an equilibrium pH of 2.22). The latter observation is consistent with the results obtained by Sole and Cole, who observed extraction of Mn(ii) only above a pH of 3.^26^ Omelchuk *et al.* observed extraction of Mn(ii) from pH 2 onwards, but these data were collected for solutions containing 1 mol L^−1^ of NaCl as a salting-out agent, and are hence not comparable with the conditions reported in our work.^27^ The data in Fig. 3 thus show that selectivity for Co(ii) and Ni(ii) over Mn(ii) is diminished in the synthetic extractant. However, at a pH of 1.69, when Mn(ii) extraction is first detected with significance, the separation factor (ratio of *D*-values) remains exceedingly high, at 3.1 × 10^5^ for Ni(ii)/Mn(ii) and 1.5 × 10^4^ for Co(ii)/Mn(ii), allowing for facile separation of Co and Ni from Mn at lower pH-values.

The discrepancy in Mn(ii) extraction between both tested extractants could either arise from the larger fraction of monothiophosphinic acid synthesized extractant formulation, or from the presence of other impurities in Cyanex 301. In the former case, MTPhos would act as a synergist in Mn(ii) extraction, while in the latter case, the other impurities would act as antagonists. Unlike the synthesized extractant, Cyanex 301 also contains significant amounts of tris(2,4,4-trimethylpentyl)phosphine sulfide (TOPS) and the oxidized disulfide derived from bis(2,4,4-trimethylpentyl)dithiophopsphinic acid. Two properties of Cyanex 301, namely gel formation at a pH over 2 and the absence of Mn(ii) extraction below a pH of 2, were not observed for any of the synthesized extractants, irrespective of their purity. Thus, the two other impurities in Cyanex 301, TOPS and the disulfide, are likely to be responsible for the emergence of these properties. The dipolar nature of TOPS, renders it the most likely to interact with extracted metal ions and metal-extractant complexes. It is worth noting that phosphine sulfides themselves have also been commercialized as extractants (*e.g.* Cyanex 471X, triisobutyl phosphine sulfide), but these are no longer available on the market.

### Slope analysis to investigate difference in extraction of Co(ii) and Ni(ii)

**Fig. 4:**
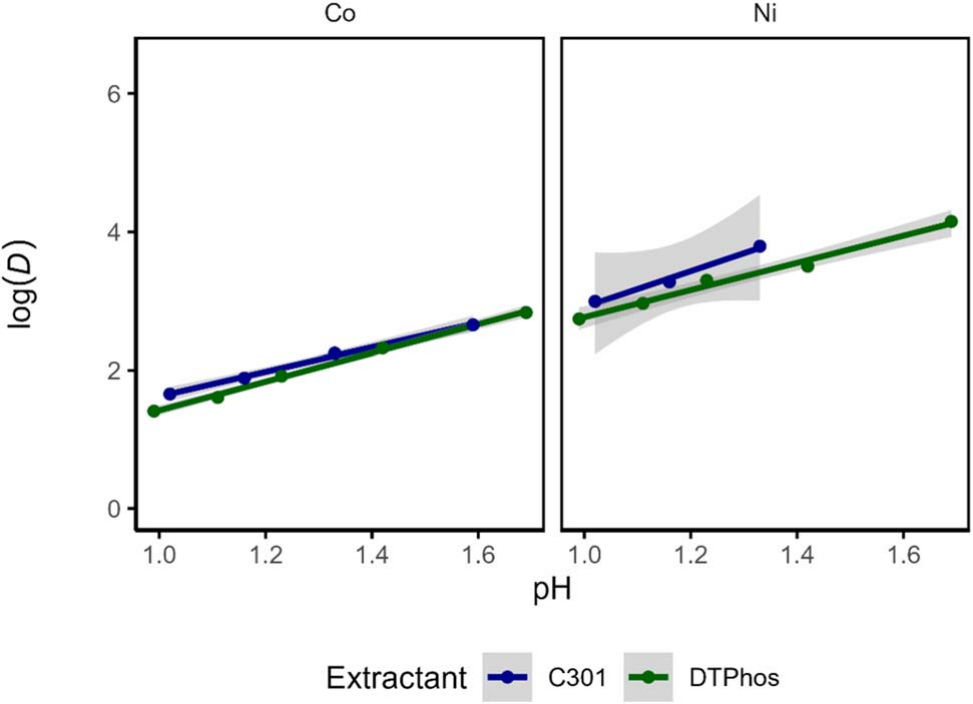
Evaluation of the logarithm (base 10) of the distribution ratio *versus* the equilibrium pH for both extractant solurions. The line is estimated *via* ordinary least squares and the grey areas represent 95% confidence intervals.

While visual comparison of the pH-extraction curves allowed investigating differences in extraction behavior of Mn(ii), it did not reveal whether there are important differences in the extraction of Co(ii) and Ni(ii), which is nearly quantitative at low pH. To assess potential differences, the performance of both extractants in Ni(ii) and Co(ii) extraction was compared by evaluating the distribution values visually, accompanied by a regression analysis of the logarithmic transformation of the distribution ratios (*D*). Since the concentrations of metals in the organic phase were not measured, the concentration in the organic phase was calculated based on the mass balance (eqn (2)). A non-linear relationship is observed for the distribution ratio as a function of pH. The curves show no obvious difference in Co(ii) extraction behavior, but show clear differences in Ni(ii) extraction behavior, with the commercial Cyanex 301 having larger *D* values. These observations are verified by inspecting the log(*D*) *vs.* pH plot, where overlap between extractants is more clear for Co(ii) than for Ni(ii) (Fig. 4 and 1).

Given the fact that for each extracted metal, two protons should be extracted, a slope analysis can be performed (Fig. 4 and 2). However, instead of performing the slope analysis on each combination of metal and extractant separately, a multiple linear regression model was used to estimate the effect of extractant and metal. To this end, the following model is estimated:

3 log(*D*) = *β*_0_ + *β*_1_ × pH + *β*_2_ × metal_*i*_ + *β*_3_ × extractant_*j*_ + *β*_4_ × metal_*i*_ × extractant_*j*_ + *ε*_*i*_

within this equation, the value of *β*_1_ provides information on the number of protons that are exchanged in the extraction mechanism, *β*_2_ provides information on the difference in log(*D*) between the two metals (*i.e.* the separation), *β*_3_ provides information on the influence of extractant used on log(*D*), and *β*_4_ provides information on the degree to which the separation of the metals is influenced by the extractant used (and *vice versa*) on the log(*D*). The final parameter, *ε*_i_, accounts for the error on individual data points.

**Fig. 5:**
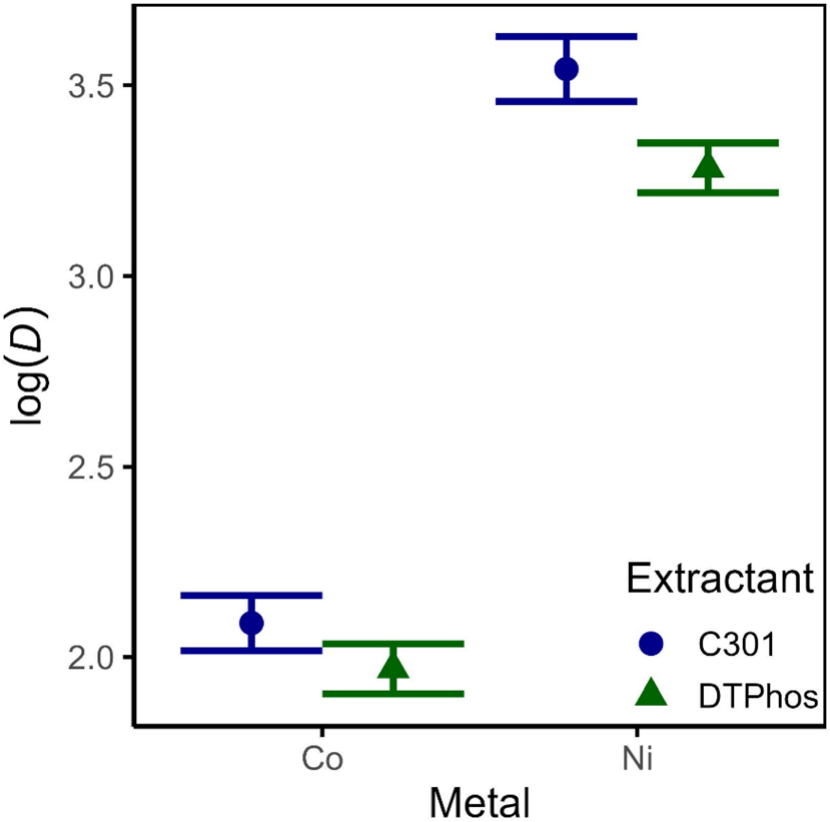
Evaluation of the multiple linear regression model. Visual comparison between the estimated logarithm (base 10) of the least squares mean distribution ratio for each combination of metal and extractant.

The analysis reveals that there is no significant difference between log(*D*) for Co(ii) between the extractants, in agreement with expectations based on the prior visual inspection, but there are significant differences for all other comparisons (*i.e.* the log(*D*) for Ni(ii) is significantly different between extractants and the log(*D*) for each extractant is significantly different between Ni(ii) and Co(ii). To summarize, the most pronounced significant differences are based on the comparison between metals. The comparison of log(*D*) between extractants for Ni(ii) is also significant, but with more uncertainty. Note that while the graph (Fig. 5) might imply a statistical interaction effect between metal and extractant (*i.e. β*_23_ ≠ 0), the observed *p*-value for this parameter is slightly above the threshold (*α* = 0.05) and hence the effect is not considered significant (Table 3), potentially due to a lack of statistical power. A nonzero value for *β*_23_ implies that different metals are affected differently by the nature of the extractant. Considering the strong difference in extraction performance of Mn(ii), it is reasonable to assume that a more extensive analysis including Mn(ii) could yield a nonzero value for *β*_23_. Lastly, the estimated number of exchanged protons by the model is *β*1 ≈ 1.99 ± 0.07 with a 95% CI [1.84, 2.16], which is in agreement with the fact that two protons should be exchanged with one Co(ii) or Ni(ii) ion. An alternative model was evaluated, which included interaction effects between pH and metal on the one hand, and pH and extractant on the other hand. As addition of these effects did not significantly improve the amount of variation in the data explained by the model, they were excluded from the model. This implies that there is no statistical evidence of a change in the mechanisms of extraction for the various metals and extractants (see SI, page S8 for ANOVA table).

**Table 3:** Estimates of the regression coefficients of the multiple linear regression model with respective standard error and hypothesis tests (H_0_: coefficient = 0, H_1_: coefficient ≠ 0). Residual standard error = 0.067 on 12 degrees of freedom. *R*^2^ = 0.99, F-statistic = 578.6 on 12 and 4 degrees of freedom, *p*-value = 0.0000. ***: < 0.001, *: < 0.05, ·: < 0.06

Parameter	Estimate	Standard error	*t* value	Pr(<|*t*|)	
Intercept	−0.437	0.099	−4.421	0.0008	***
pH	1.999	0.073	27.367	0.0000	***
Metal (Ni)	1.453	0.052	28.196	0.0000	***
Extractant (DTPhos)	−0.120	0.045	−2.672	0.0203	*
Metal (Ni) * extractant (DTPhos)	−0.139	0.067	−2.088	0.0588	·

To conclude, the analysis reveals that the synthesized DTPhos performs worse in terms of separation compared to Cyanex 301, but does not result in gel formation at a higher percentage of saponification. Furthermore, despite the observed difference, both mixtures are able extract Co(ii) and Ni(ii) nearly quantitatively with a single contact. Since the high fraction of MTPhos in the synthesized DTPhos might be one of the causes of this difference, it was decided to enrich the synthesized solutions in DTPhos and repeat the extraction experiment.

### Effect of DTPhos enrichment on extraction behaviour in sulfate and MSA media

**Fig. 6:**
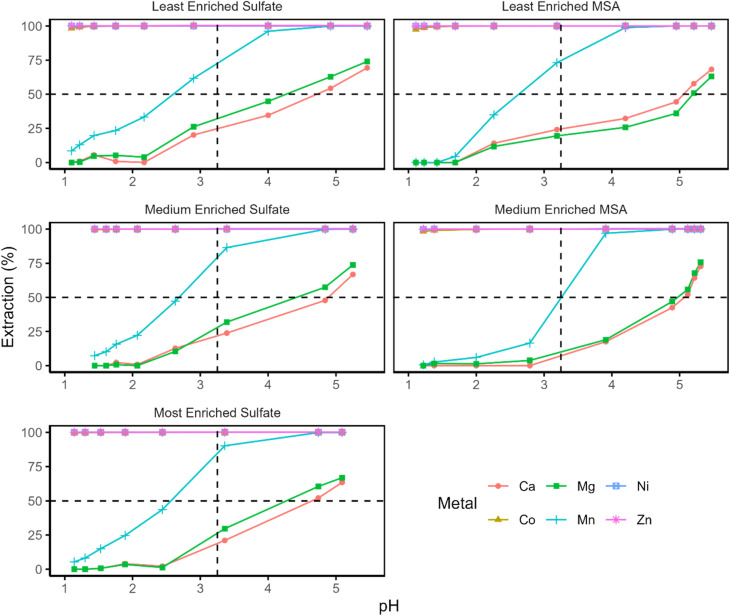
Equilibrium pH-extraction curves of the different metals extracted using the synthesized DTPhos mixtures at different levels of enrichment (30 vol%) in methanesulfonate (MSA) or sulfate medium. Composition of the aqueous phase can be found in Table 1.

In order to distinguish the effect of the MTPhos impurity from that of the TOPS and disulfide impurities from, the three synthesized extractant formulations were mutually compared, each with varying concentrations of MTPhos impurity, but no TOPS or disulfide impurities (Fig. 6). Additionally, both sulfate and methanesulfonate feed solutions were compared, as the aqueous anion may also influence extraction behavior. Importantly, third-phase formation was never observed in the methanesulfonate systems, while it was consistently observed in sulfate systems at elevated pH.

Under the low loading conditions (<50%) investigated in the current assay, the presence of MTPhos impurities does not appear to affect the extraction of Mn(ii) to a significant extent at an equilibrium pH above 2. This is evident upon visual inspection of the graphs of the sulfate systems. At first sight, the extraction curve appears to shift towards higher pH in the methanesulfonate systems upon increasing the purity of the extractant. However, this appears to be entirely contingent on a single data point at a pH of about 3, and no further data at intermediate percentages extraction were obtained. We thus do not consider this result to be significant. At lower pH (<2), by contrast, higher percentages extraction are consistently Mn(ii) in sulfate systems than in methanesulfonate systems obtained for. While there is insufficient evidence available to draw any concrete conclusions, this observation may either result from coordination of sulfate in the extracted species at lower pH, or it may be due to a salting-out effect caused by the strongly hydrated sulfate anion.^28^

**Fig. 7:**
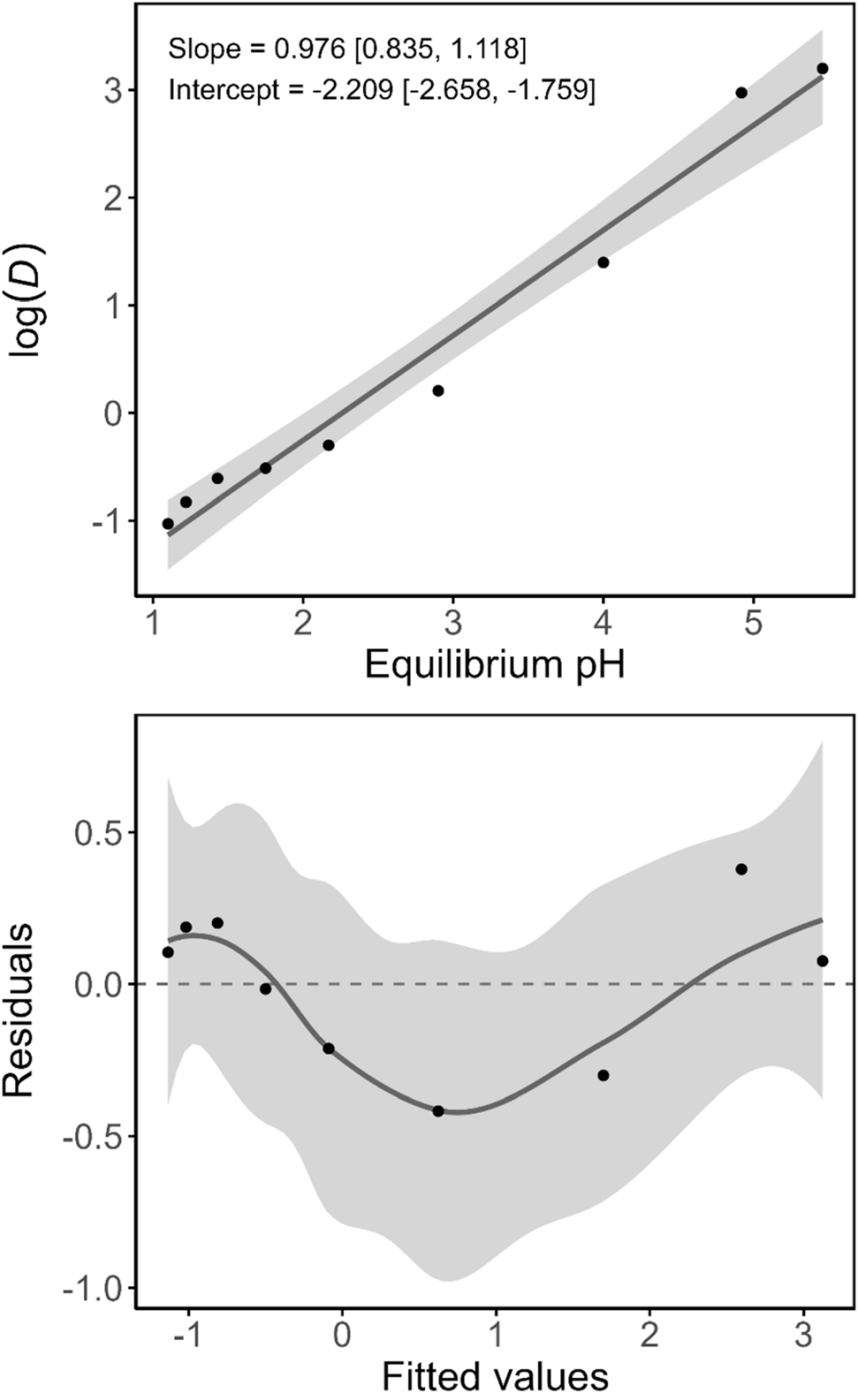
Top: logarithm (base 10) of the distribution ratio of Mn(ii) in the least-enriched DTPhos, sulfate system as a function of the equilibrium pH. Linear regression line and 95% confidence band are estimated using ordinary least squares. Slope and intercept are given with the respective 95% confidence intervals. Bottom: plot of the residuals with locally estimated scatterplot smoothing (LOESS) regression line with 95% confidence bands as a guide for the eye.

The pH-extraction curves also deviate strongly from their expected sigmoidal shape in the sulfate systems, indicating a shift in the mechanism or in the solvation/aggregation state of the extracted species. Further information was obtained by performing a slope analysis of Mn(ii) for the synthetic extractant mixtures (Fig. 7 and SI, page S9). The residuals of obtained log–log curves exhibited strong and consistent patterns, indicating deviation from linearity, which were evident if sufficient data points were available. The consistency of these patterns precludes any inference of randomness. Moreover, the slope of the linear regression through the data was approximately equal to 1 for every tested combination of extractant and feed solution (Table S2). There were no large differences between the slope of the curves for the various samples, taking into account the uncertainty on their estimation. A slope of 1 for log(*D*) as a function of pH is highly unexpected for extraction of a divalent metal ion by an acidic extraction, as two protons are expected to be exchanged in order to maintain charge neutrality of the phases. One may postulate an extraction mechanism involving coextraction of sulfate or methanesulfonate anions. However, this appears unlikely, as FT-IR spectra of the loaded organic phase presented no evidence of the presence of sulfate in the organic phase after loading the organic phase from a Mn(ii) sulfate solution (25% loading). Alternatively, one may envision the extraction of Mn(OH)^+^, but this species is only expected to be formed in very low concentrations at a pH greater than 10.5.^29^ Considering the poor linearity of the data, it hence appears likely that the mechanism of extraction shifts with increasing pH. Such a shift could involve, for instance, a change in the aggregation or solvation state of the extracted species, or a change in the nature of the extracted species itself. Changes in speciation as a function of loading or concentration been described for other acidic extractants.^30,31^

Given the limited influence of the MTPhos concentration on the extraction behavior of Mn(ii), the higher selectivity of commercial Cyanex 301 for Co(ii) and Ni(ii) over Mn(ii) appears to be the result of antagonism induced by one of the other impurities present in the commercial formulation. The most likely candidate is TOPS, due to its relatively high concentration in the formulation and its high propensity to interact with metal ions and extractant molecules. This phenomenon is reminiscent of the comparison between Cyanex 272 (containing a significant amount of trioctylphosphine oxide impurity) and Ionquest 290 (lacking said impurity), where Cyanex 272 has been reported exhibit a higher selectivity for Co(ii) over Ni(ii) than Ionquest 290.^32^

For Ca(ii) and Mg(ii), extraction appears to be similarly unaffected by the presence of MTPhos impurities in the organic phase. Again, extraction in the intermediate pH range (2–5) appears to be lower only in the methanesulfonate system using the medium-purity extractant. However, the difference is mainly located in a single data point at a pH of approximately 3. Considering the higher scatter on the Mg(ii) and Ca(ii) extraction data, the curves do not appear to be significantly different from those for the other investigated systems.

### Statistical comparison between extraction behavior

**Fig. 8:**
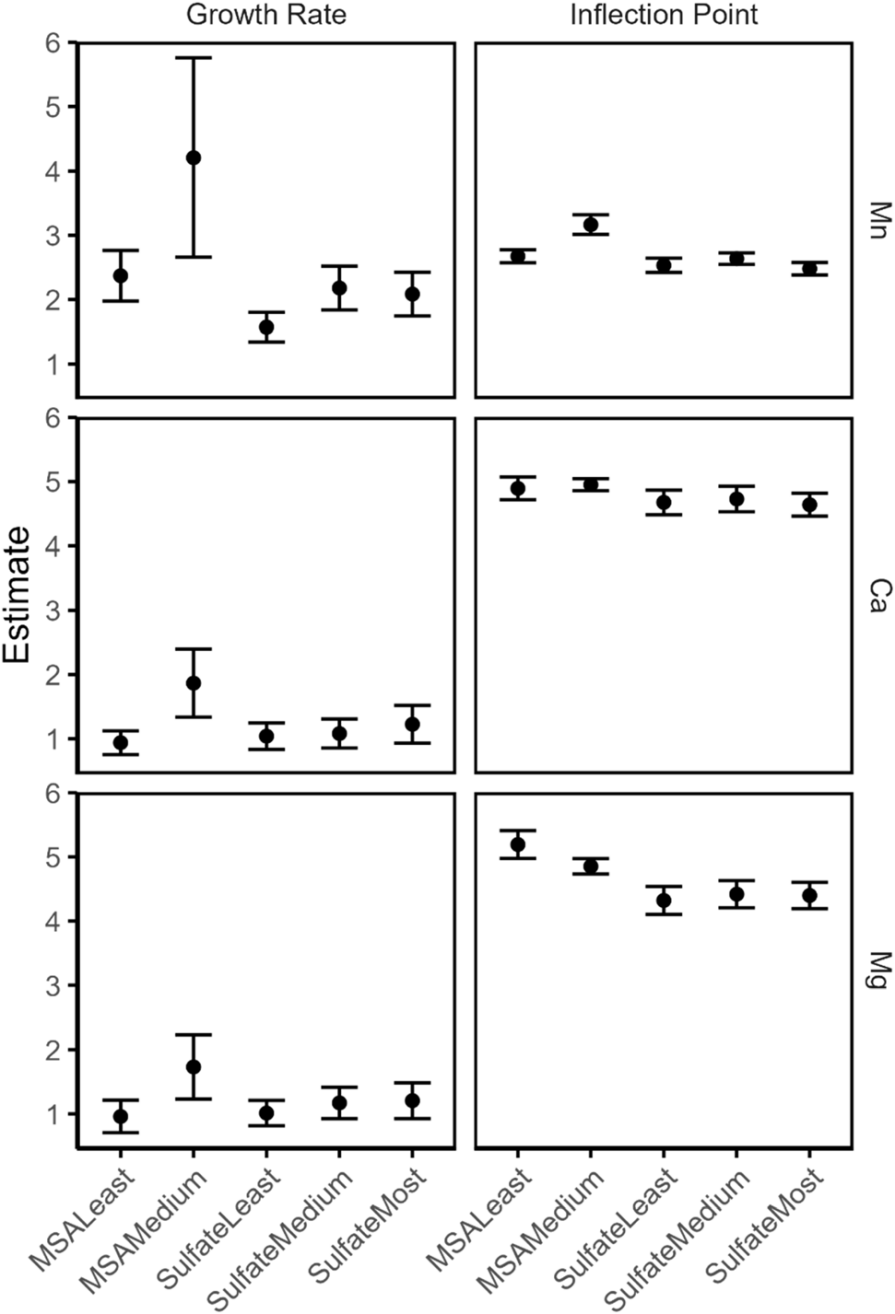
Plot of the estimated parameters (with 95% confidence intervals) after fitting a logistic regression model (eqn (3)) to the extraction data.

Since one of the goals of this comparison is to evaluate the effect of the impurities on the extraction of undesired metals such as Mn(ii), Ca(ii) and Mg(ii), it was decided to formally compare the extraction behavior of Mg(ii), Mn(ii) and Ca(ii) by estimating the pH_50_ and growth rate through fitting logistic functions to the data (eqn (4)). This equation arises from the general extraction equation (see ESI, page S8) and is of the following form:4
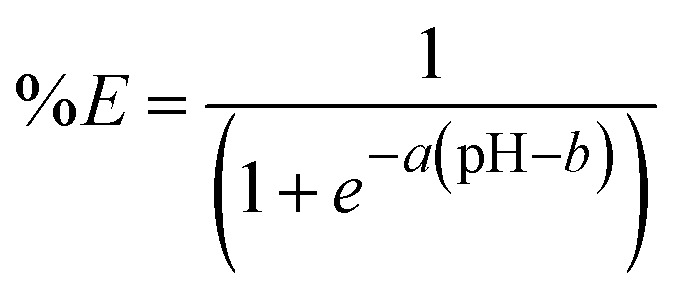
where the estimated parameters are: *a* (growth rate) and *b* (inflection point). The growth rate parameter corresponds to the gradient of the curve, and is dependent on the number of protons exchanged per extracted metal ion. Within the context of extraction, the estimate of the inflection point is an estimate of the pH_50_. The equation implies symmetry around the inflection point, with an upper asymptote of 100% extraction and a lower asymptote of 0% extraction. After estimating the parameters for the three metals, the effect of the medium (*i.e.* sulfate *vs.* methanesulfonate) and enrichment of DTPhos (*i.e.* least, medium and most) the estimates are graphically compared (Fig. 8). No significant differences in the extraction behavior of any of the metals were observed in sulfate medium. In methanesulfonate medium, the growth rate was significantly higher (95% CL) at higher DTPhos purity for Ca(ii), while the inflection point was significantly (95% CL) higher for Mn(ii) and lower for Mg(ii). However, uncertainty on the experimental data points are not taken into account, and the experimental curves systematically deviate markedly from their expected sigmoidal shapes (*vide supra*). Hence, the uncertainty on the estimates may be higher than predicted by the statistical model.

## Conclusions

Bis(2,4,4-trimethylpentyl)dithiophosphinic acid (DTPhos) can be prepared in multi-gram scale from the corresponding commercial phosphinic acid extractants using the Bergman reagent, generated *in situ* using the Berzelius reagent (P_4_S_10_). The synthesized DTPhos mixtures demonstrated efficient extraction of Co(ii), Ni(ii), and Zn(ii), even in the absence of saponification. Notably, no third-phase formation was observed in methanesulfonate (MSA) systems, indicating a distinct operational advantage over Cyanex 301. Although based Ni(ii) extraction was initially somewhat reduced in the crude DTPhos formulation, Co(ii) and Zn(ii) performance remained high across all conditions. Enrichment of DTPhos led to subtle, but measurable changes in extraction behavior, as confirmed by estimating the sigmoidal extraction pattern and performing a slope analysis. While deviations from expected stoichiometry and mechanistic shifts were observed, especially in sulfate media, these effects did not significantly compromise separation efficiency. Overall, the results highlight the robustness of the synthesized DTPhos system, highlighting its potential as a practical lab-scale solution for selective metal extraction in acidic media.

## Conflicts of interest

There are no conflicts to declare.

## Supplementary Material

RA-016-D5RA06902E-s001

## Data Availability

The data supporting this article have been included as part of the supplementary information (SI). Supplementary information: details on chemicals, reagents and instrumentation, full synthetic procedure for DTPhos, details on extractant composition, details on statistical analysis, and NMR and FT-IR spectra, slope analysis of Mn(ii) extraction, which should be added to the enumeration. See DOI: https://doi.org/10.1039/d5ra06902e.
