# Supplementary material for: Effect of monothiophosphinic acid and phosphine sulfide impurities on the solvent extraction behavior of bis(2,4,4-trimethylpentyl)dithiophosphinic acid from sulfate and methanesulfonate media
Source: RSC Adv. 2026 Jan 5;16(2):1357–67. doi: 10.1039/d5ra06902e (PMC12766893; doi:10.1039/d5ra06902e)
Supplement: RA-016-D5RA06902E-s001 [file RA-016-D5RA06902E-s001.pdf]

Supplementary Information (SI) for RSC Advances.
This journal is © The Royal Society of Chemistry 2025

Electronic Supplementary Information for:

Effect of Monothiophosphinic Acid and Phosphine Sulfide Impurities on the Solvent Extraction Behavior of Bis(2,4,4-trimethylpentyl)dithiophosphinic Acid from Sulfate and Methanesulfonate Media

Tim Balcaen[1], Koen Binnemans[1,] Stijn Raiguel[1*]

[†] KU Leuven, Department of Chemistry, Celestijnenlaan 200F, P.O. box 2404, B-3001 Leuven, Belgium.

*Corresponding author:

Email: stijn.raiguel@kuleuven.be

Table of contents

NMR Spectra of synthesized bis(2,4,4-trimethylpentyl)dithiophosphinic acid S3

Extractant composition S4

Synthesized dithiophosphinic acid (least enriched) S5

Synthesized dithiophosphinic acid (medium enriched) S5

Synthesized dithiophosphinic acid (most enriched) S5

Commercial Cyanex 301 S6

Example: Prediction of LogD using the regression coefficients found in Fig. 5 S7

Comparison of the full second-order factorial model with the reduced model S8

Derivation of the sigmoidal function as a model for the percentage of extraction S8

Slope analysis of Mn(II) for the synthetic extractant mixtures S9

NMR Spectra of synthesized bis(2,4,4-trimethylpentyl)dithiophosphinic acid

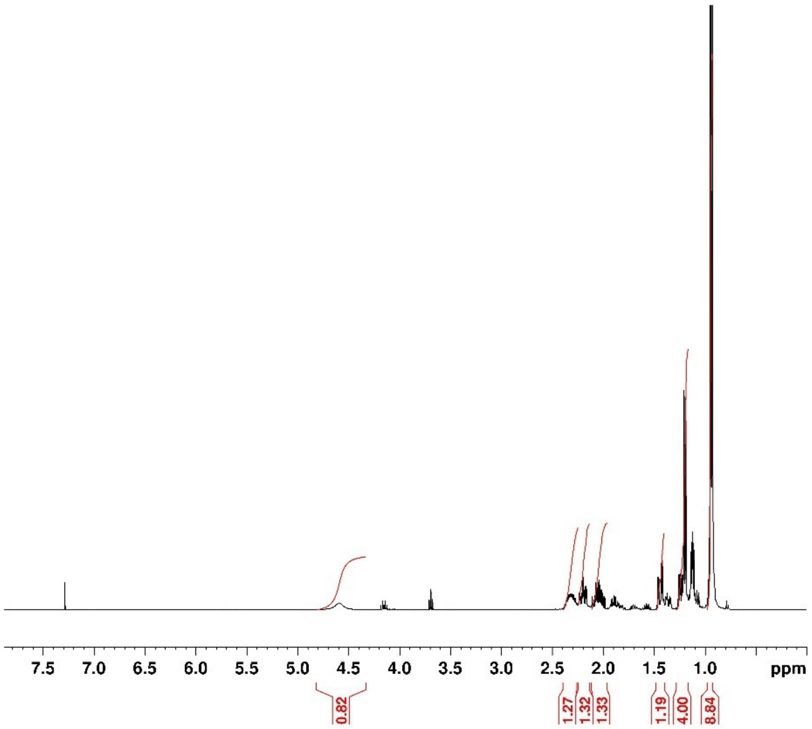

Fig. S1 – ^{1}H NMR spectrum of the most DTPhos-enriched mixture in $CDCl_3$. Traces of n-butanol and water are present within the sample.

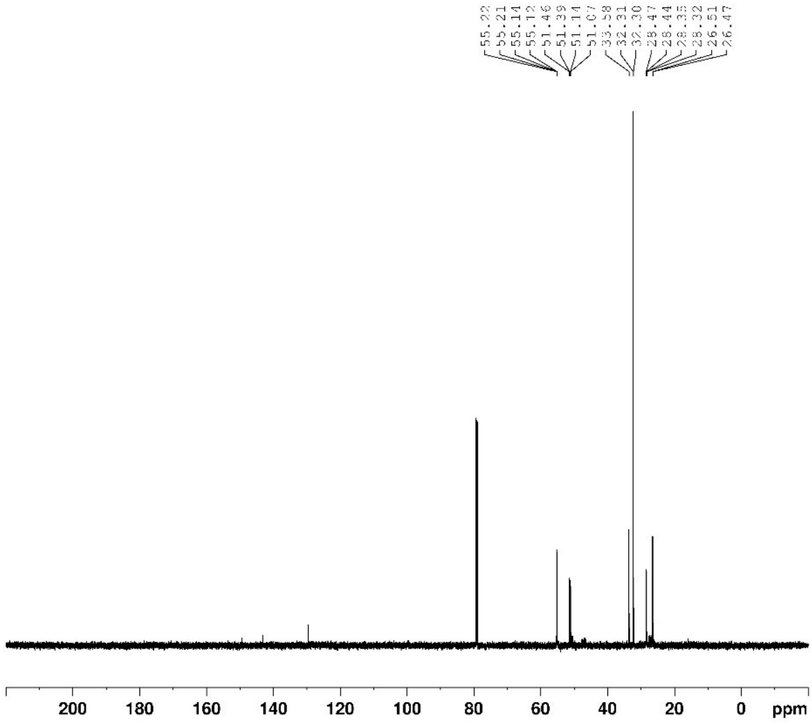

Fig. S2 – ^{13}C NMR spectrum of the most DTPhos-enriched mixture in $CDCl_3$.

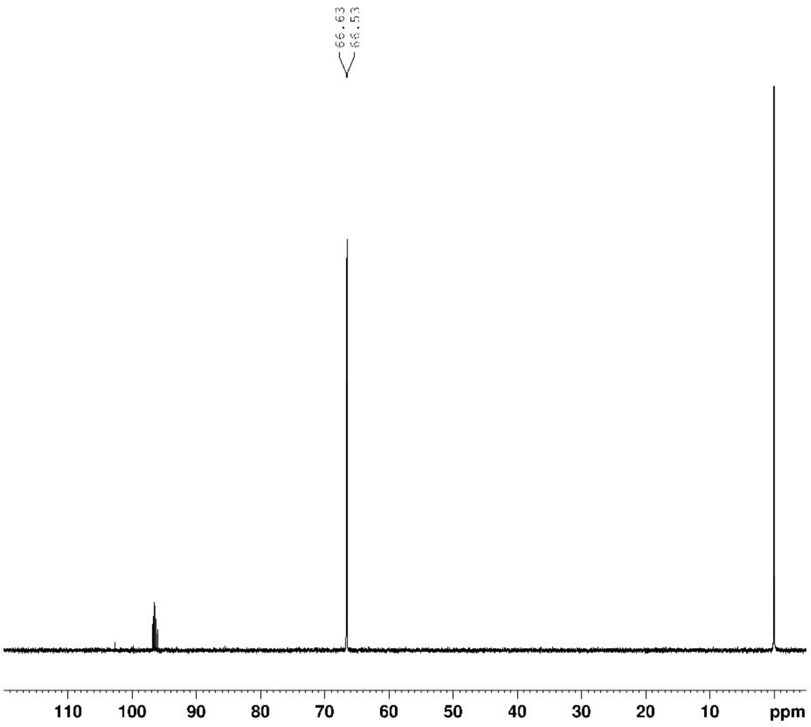

Fig. S3 – ^{31}P NMR spectrum of the most DTPhos-enriched mixture in $CDCl_3$ with 10% H_3PO_4 in D_2O as external reference.

Extractant composition

An approximate composition of the extractant solutions was determined based on the ^{31}P NMR spectrum (taking into account that the disulfide impurity contains two equivalent phosphorus nuclei). Note that this might differ from the true extractant fraction, since spin-lattice relaxation (T_1) values for the components were not determined, and the possibility exists that all nuclei do not fully relax within the applied recycle delay time (D1) of 30 s. Spectra of Cyanex 301 and the least enriched DTPhos mixture with annotations are added. For accuracy purposes, solutions were prepared on a mass basis. Densities of the extractants are based on Nguyen *et al.* (V. N. H. Nguyen, T. H. Nguyen and M. S. Lee, *Metals,* **2020**, *10*, 1105).

Synthesized dithiophosphinic acid (least enriched)

An extractant solution containing 30 V% synthesized DTPhos (least enriched) mixture was prepared by adding 3.6 mL of extractant mixture and diluted to a total volume of 12 mL with *n*-dodecane. The

synthesized DTPhos mixture was weighed at 3.4434 g (≈ 3.625 mL, given a density of 0.95 g/mL). Next, 6.243 g (≈ 8.324 mL, given a density of 0.75 g/mL) of *n*-dodecane was weighed. Together, this gives a total volume of approximately 11.95 mL. This information combined gives 0.288 g of synthesized DTPhos mixture·mL^{-1}, with approximately 68 mol% DTPhos and 29 mol% MTPhos.

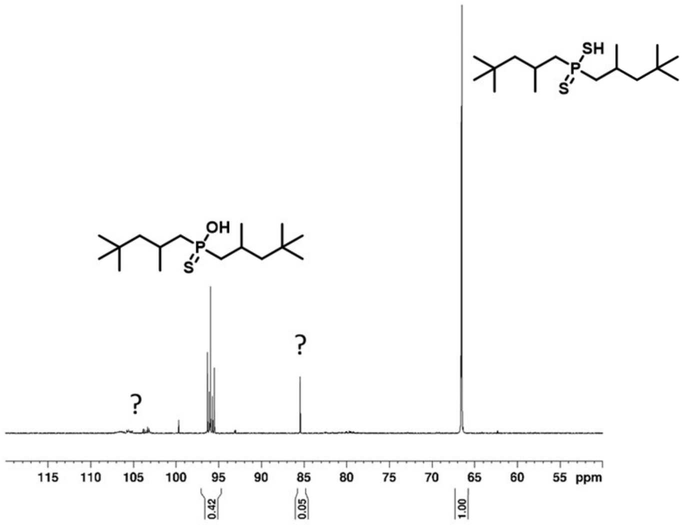

Figure S4 – ^{31}P NMR spectrum the least enriched synthesized DTPhos mixture calibrated with an external reference of 10% H_3PO_4 in D_2O.

Synthesized dithiophosphinic acid (medium enriched)

An extractant solution containing 30 V% synthesized DTPhos (medium enriched) mixture was prepared by adding 7.2 mL of extractant mixture and diluted to a total volume of 24 mL with *n*-dodecane. The synthesized DTPhos mixture was weighed at 6.8474 g (≈ 7.208 mL, given a density of 0.95 g/mL). Next, 12.4651 g (≈ 16.62 mL, given a density of 0.75 g mL^{-1}) of *n*-dodecane was weighed. Together, this gives a total volume of approximately 23.83 mL. This information combined gives 0.287 g of synthesized DTPhos mixture·mL^{-1}, with approximately 85 mol% DTPhos and 14 mol% MTPhos.

Synthesized dithiophosphinic acid (most enriched)

An extractant solution containing 30 V% synthesized DTPhos (medium enriched) mixture was prepared by adding 3.6 mL of extractant mixture and diluted to a total volume of 12 mL with *n*-dodecane. The synthesized DTPhos mixture was weighed at 3.4226 g (≈ 3.603 mL, given a density of 0.95 g/mL). Next,

6.2304 g (≈ 8.307 mL, given a density of 0.75 g/mL) of *n*-dodecane was weighed. Together, this gives a total volume of approximately 11.91 mL. This information combined gives 0.289 g·mL^{-1} of synthesized DTPhos mixture, with approximately 90 mol% DTPhos and 9 mol% MTPhos.

Commercial Cyanex 301

No pretreatment was performed on the commercial Cyanex 301. Cyanex 301 still contains significant amounts of tris(2,4,4-trimethylpentyl)phosphine sulfide (TOPS) and the oxidized disulfide byproduct. Both products might have an effect on the third-phase formation and extraction performance of the extractant mixture. Since the extractant fraction is substantially lower in Cyanex 301 as compared to the synthesized formulations, the volume percentage of Cyanex 301 was increased from 30 V% to 41 V%. To this end 5.2823 g of Cyanex 301 (≈ 5.56 mL, given a density of 0.95 g mL^{-1}) was mixed with 6.1529 g of *n*-dodecane (≈ 8.204 mL, given a density of 0.75 g mL^{-1}). Together, this gives a total volume of approximately 13.76 mL. This information combined gives 0.384 g·mL^{-1} of Cyanex 301, with approximately 68 mol% DTPhos and 4 mol% MTPhos.

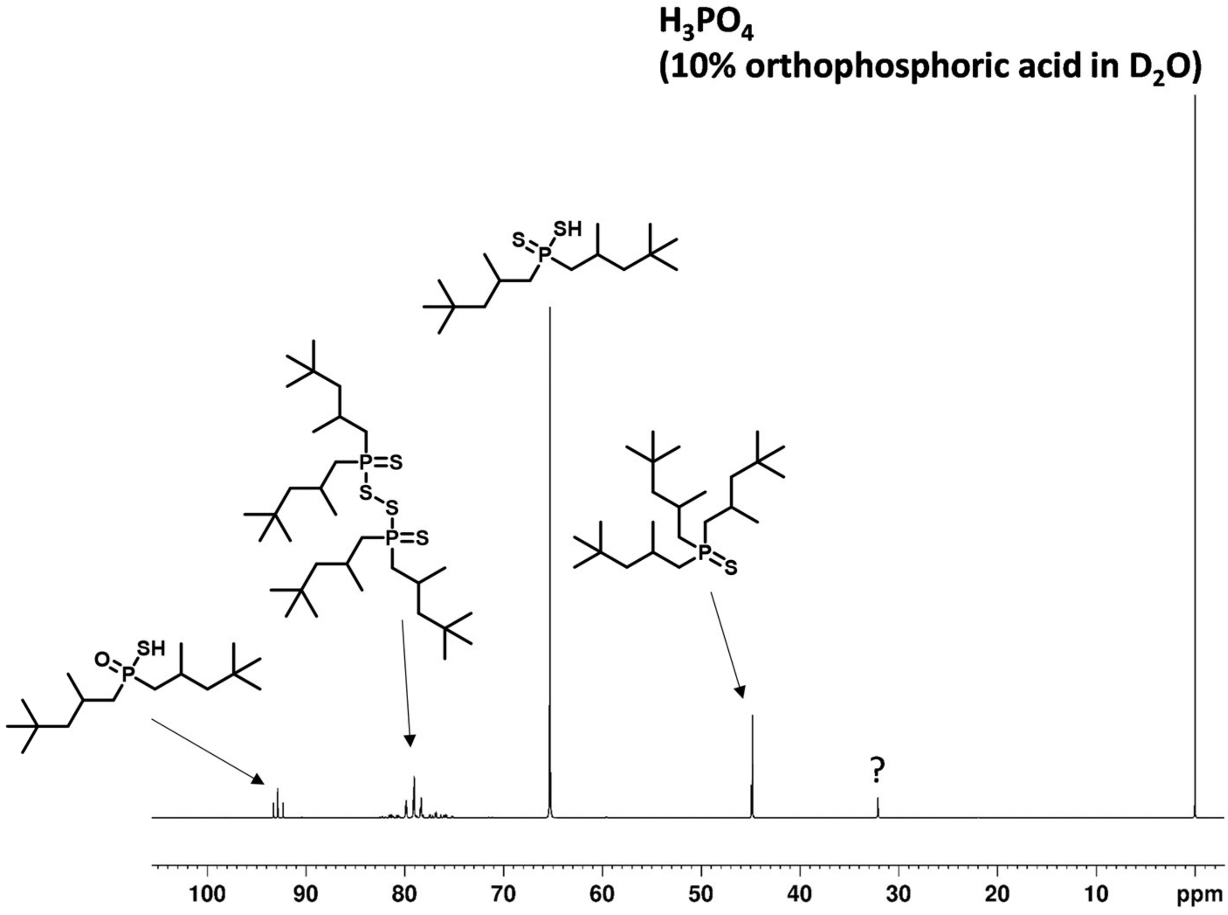

Fig. S5 – ^{31}P NMR spectrum the commercial Cyanex 301 mixture calibrated with an external reference of 10% H_3PO_4 in D_2O.

Example: Prediction of logD using the regression coefficients found in Fig. 5

To compute point predictions or interpret the effect of individual predictors from the regression model (Fig. 5, main text and **Error! Reference source not found.**), one can directly substitute values into the linear predictor using the estimated coefficients. It is important to account for the categorical variable coding used in R, which applies treatment (dummy) coding by default. For binary factors, one level serves as the reference category (e.g., Metal = Co is the reference for the Metal factor).

Table S1 – Estimates of the regression coefficients of the multiple linear regression model with respective standard error and hypothesis tests (H_0: coefficient = 0, H_1: coefficient ≠ 0). Residual standard error = 0.067 on 12 degrees of freedom. R^2 = 0.99, F-statistic = 578.6 on 12 and 4 degrees of freedom, p-value = 0.0000. ***: < 0.001, *: < 0.05, ·: < 0.06. This table is copied from the main text (Table 3).

Parameter	Estimate	Standard Error	*t* value	Pr(<|*t*|)	
**Intercept**	-0.437	0.099	-4.421	0.0008	***
**pH**	1.999	0.073	27.367	0.0000	***
**Metal (Ni)**	1.453	0.052	28.196	0.0000	***
**Extractant (DTPhos)**	-0.120	0.045	-2.672	0.0203	*
**Metal (Ni) * Extractant (DTPhos)**	-0.139	0.067	-2.088	0.0588	·

For example, to estimate log*D* when Extractant = C301, Metal = Co, and pH = 1.26 (= the average pH), the prediction is:

$$E[logD] = \hat{\beta}_0 + \hat{\beta}_1 * pH + \hat{\beta}_2 * Co + \hat{\beta}_3 * C301 + \hat{\beta}_4 * Co * C301 \quad \textit{(Eq. S1)}$$

$$E[logD] = -0.437 + 1.999 * 1.26 + 1.435 * 0 - 0.120 * 0 - 0.139 * 0 * 0 = 2.08 \quad \textit{(Eq. S2)}$$

Performing the same calculation, but for Metal = Ni, the following is obtained:

$$E[logD] = \hat{\beta}_0 + \hat{\beta}_1 * pH + \hat{\beta}_2 * Ni + \hat{\beta}_3 * C301 + \hat{\beta}_4 * Ni * C301 \quad \textit{(Eq. S3)}$$

$$E[logD] = -0.437 + 1.999 * 1.26 + 1.435 * 1 - 0.120 * 0 - 0.139 * 1 * 0 = 3.52 \quad \textit{(Eq. S4)}$$

This approach can be used to interpret the model's behavior across all combinations of categorical and continuous predictors.

Comparison of the full second-order factorial model with the reduced model

The following two nested models were compared using the “ANOVA” function from base R.

$$Reduced: log(D) = \beta_0 + \beta_1 * pH + \beta_2 * Metal_i + \beta_3 * Extractant_j + \beta_4 * Metal_i * Extractant_j + \varepsilon_i \quad \textit{(Eq. S5)}$$

$$Full: log(D) = \beta_0 + \beta_1 * pH + \beta_2 * Metal_i + \beta_3 * Extractant_j + \beta_4 * Metal_i * Extractant_j + \beta_5 * Metal_i * pH + \beta_6 * pH * Extractant_j + \varepsilon_i \quad \textit{(Eq. S6)}$$

The results of the test are shown in Table 1. These results reveal that the reduced model does not perform significantly worse compared to the full model and hence it is allowed to apply the reduced model.

Table S2 – ANOVA table of the comparison between the full and reduced models.

Model	Residual df	Residual sum of squares	Df	Sum of squares	F	Pr(>F)
Reduced	12	0.053				
Full	10	0.051	2	0.002	0.225	0.802

Derivation of the sigmoidal function as a model for the percentage of extraction

The extraction equilibrium can be represented as follows.

$$M^{n+} + (n+a)HA \overset{K_{ex}}{\leftrightharpoons} nH^{+} + MA_nHA_a \#(Eq.S7)$$

By writing the formula for the equilibrium constant and substituting the value for the distribution coefficient, the following equation is obtained.

$$K_{ex} = \frac{[\bar{MA_n}\bar{HA_a}][H^{+}]^{n}}{[M^{n+}][\bar{HA}]^{(n+a)}} = D\frac{[H^{+}]^{n}}{[\bar{HA}]^{(n+a)}} \#(Eq.S8)$$

Substituting this in the equation for the percentage of extracted metal, the following equation is obtained, assuming the volume ratio of the organic and aqueous phase equals 1.

$$\%E = \frac{D}{D+1} = \frac{K_{ex}[\bar{HA}]^{(n+a)}[H^{+}]^{-n}}{K_{ex}[\bar{HA}]^{(n+a)}[H^{+}]^{-n}+1} = \frac{1}{1+\frac{[H^{+}]^{n}}{K_{ex}[\bar{HA}]^{(n+a)}}}$$

$$= \frac{1}{1+\frac{1}{K_{ex}[\bar{HA}]^{(n+a)}}10^{-n*pH}} \#(Eq.S9)$$

This last equation has a sigmoidal shape and can reformulated such that it matches the expression in the main text. If a = n·ln10 and b = -(1/n)log$_{10}$(K$_{ex}$·$[\bar{HA}]^{(n+a)}$) then the equation simplifies to the following.

$$\%E = \frac{1}{1 + e^{-a(pH - b)}} \#(Eq. S10)$$

Slope analysis of Mn(II) for the synthetic extractant mixtures

Fig. S7 shows the residuals of the log-log curves after slope analysis focusing on Mn(II) for all synthetic extractant mixtures. The data for the least enriched DTPhos formulation in MSA were omitted, as too few data points (*i.e.* 4 data points) were available to perform a visual inspection of the residuals (Fig. S7). Additionally, the slope and intercept and corresponding confidence intervals of the slope analyses are given in Table S2.

Table S3 – Slopes and intercepts linked to the slope analysis of the Mn(II) isotherms corresponding to the synthetic extractant mixtures (with the exception of the least enriched mixture in MSA medium).

group	intercept	95% CI on intercept	slope	95% CI on slope
SulfateLeast	-2.209	[-2.658, -1.759]	0.976	[0.835, 1.118]
SulfateMedium	-2.916	[-3.215, -2.617]	1.188	[1.093, 1.283]
SulfateMost	-2.644	[-2.979, -2.309]	1.151	[1.041, 1.260]
MSAMedium	-3.722	[-4.382, -3.062]	1.359	[1.182, 1.537]

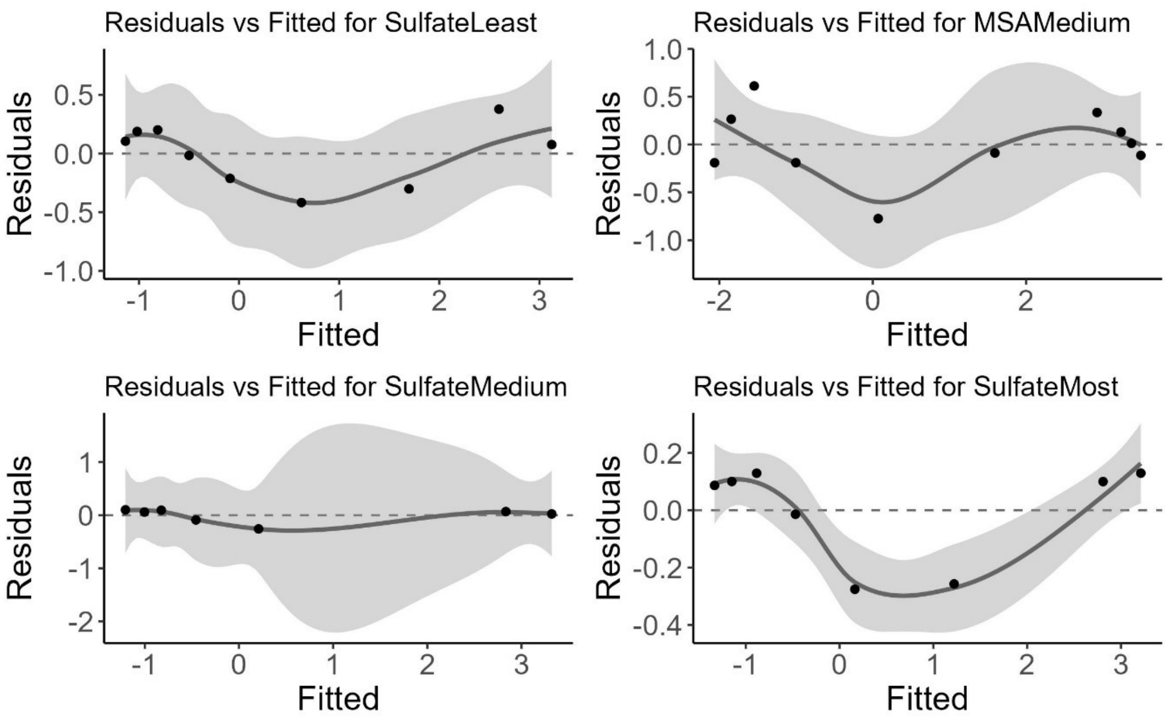

Fig. S6 – Residual plots after performing slope analysis (i.e. simple linear regression) on the log-log curves of the Mn(II) extraction isotherms for the different synthetic extractant mixtures. MSALeast is not included, since insufficient data points were available to perform slope analysis. Locally estimated scatterplot smoothing (LOESS) regression lines are added with 95% confidence bands as guides for the eye.

Fig. S8 shows the FT-IR spectrum of the most enriched DTphos formulation (30 vol%) after extraction of Mn(II) from sulfate medium (25% loaded). This spectrum is compared to the unloaded organic phase after equilibration of water. An increase in the intensity of the O-H stretching (approx. 3350 cm^{-1}) and scissoring (approx. 1650 cm^{-1}) bands is observed upon loading, demonstrating a strong increase in the equilibrium water content. However, no new bands arise in the area in which the sulfate antisymmetrical stretching vibration is expected (1050-1150 cm^{-1}). This indicates that no significant amounts of sulfate are coextracted along with Mn(II).

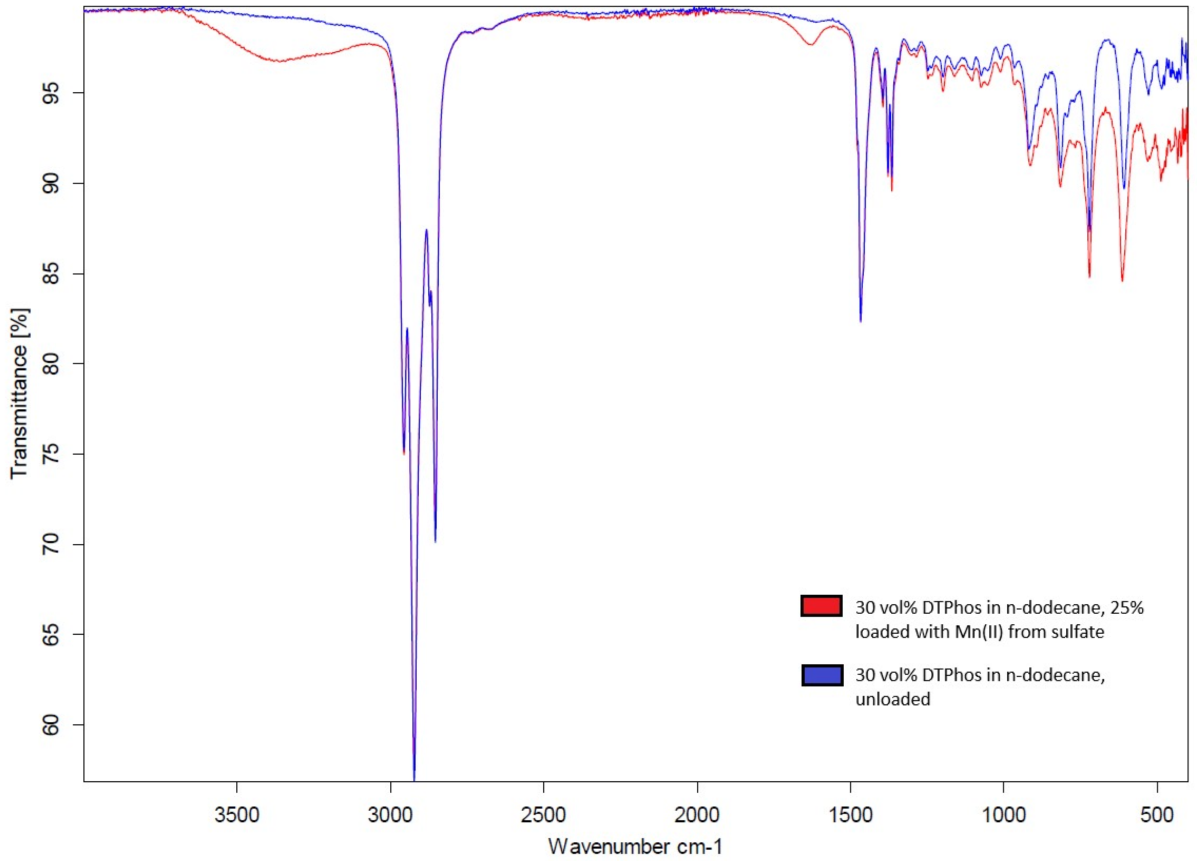

Fig. S7 – FT-IR spectra of 30 vol% DTPhos (most enriched) in *n*-dodecane, comparing unloaded (blue) vs. 25% loading of Mn(II) from sulfate medium.
